# Effect of *Euterpe oleracea* Mart. (Açaí) Seed Bioproducts on *Trypanosoma cruzi*

**DOI:** 10.3390/biology15010096

**Published:** 2026-01-02

**Authors:** Henrique Previtalli-Silva, Daiana de Jesus Hardoim, Raphael de Lucena Banaggia, Carla J. Moragas-Tellis, Paulo Victor Ramos de Souza, Maria Dutra Behrens, Thiago de Souza Dias Silva, Kátia da Silva Calabrese, Flávia de Oliveira Cardoso

**Affiliations:** 1 Laboratory of Protozoology, Oswaldo Cruz Institute, FIOCRUZ, Av. Brasil, 4.365, Manguinhos, Rio de Janeiro 21040-360, Brazil; henriqueprevitalli89@gmail.com (H.P.-S.); hardoim@ioc.fiocruz.br (D.d.J.H.); raphaelbanaggia@gmail.com (R.d.L.B.); 2 Postgraduate Program in Tropical Medicine, Oswaldo Cruz Institute, FIOCRUZ, Av. Brasil, 4.365, Manguinhos, Rio de Janeiro 21040-360, Brazil; 3 Laboratory of Natural Products for Public Health, Pharmaceutical Technology Institute (Farmanguinhos), FIOCRUZ, R. Sizenando Nabuco, 100, Manguinhos, Rio de Janeiro 21040-360, Brazil; carla.tellis@fiocruz.br (C.J.M.-T.); paulo.ramos@fiocruz.br (P.V.R.d.S.); maria.behrens@fiocruz.br (M.D.B.); 4 Department of Organic Chemistry, Chemistry Institute, Federal Rural University of Rio de Janeiro, Seropédica 23890-000, Brazil; thiago.diassilva87@gmail.com

**Keywords:** Chagas disease, *Euterpe oleracea*, *Trypanosoma cruzi*, treatment, Açaí

## Abstract

Chagas disease remains a major public health challenge due to the limited effectiveness and considerable side effects of existing treatments, particularly during the chronic stage. Açaí (*Euterpe oleracea*) seeds have gained increasing attention as a source of bioactive compounds with potential therapeutic applications. In this study, hydroalcoholic extracts and solvent fractions obtained from açaí seeds were chemically characterized by ESI/MS and HPLC–MS/MS and evaluated for their cytotoxicity and antiparasitic activity against different developmental stages of *Trypanosoma cruzi* (Y strain). Chemical profiling revealed a predominance of phenolic compounds, particularly catechins and procyanidins, which were identified as major constituents of the hydroalcoholic extract and the ethyl acetate fraction. Cytotoxicity assays performed on murine peritoneal macrophages demonstrated low toxicity, with CC_50_ values exceeding 500 µg/mL for most samples, indicating a favorable in vitro safety profile. Antiparasitic assays showed weak activity against epimastigote forms; however, significant inhibitory effects were observed against bloodstream trypomastigotes, cell culture-derived trypomastigotes, and intracellular amastigotes. Notably, the hydroalcoholic extract exhibited the highest selectivity against intracellular amastigotes, with a selectivity index greater than 10, fulfilling key criteria proposed by the Drugs for Neglected Diseases initiative (DNDi) for early-stage hit compounds. Flow cytometry analysis showed that both the hydroalcoholic extract and the ethyl acetate fraction induced parasite cell death through late apoptosis-like and necrosis. Together, these findings highlight the antiparasitic potential of *E. oleracea* seed extracts, particularly against clinically relevant stages of *T. cruzi*, and support further investigation of these bioproducts as promising candidates for the development of new therapeutic strategies for Chagas disease.

## Simple Summary

Chagas disease is a serious infection caused by a microscopic parasite transmitted by insects and current treatments are often ineffective and cause many side effects, especially for people who are already in the long-term stage of the disease. Therefore, there is an urgent need to discover safer and more effective substances. In this study, we investigated natural compounds found in the seeds of the açaí fruit, a widely consumed product in Brazil whose seeds are usually discarded. These seeds contain molecules with known antioxidant and anti-inflammatory properties, which may help reduce infection-related damage. We tested different extracts from the seeds on several stages of the parasite that causes Chagas disease. The compounds showed a strong ability to reduce the parasite population while remaining harmless to healthy immune cells. We also discovered that these compounds could trigger a process that leads the parasite to die in a controlled way. These findings indicate that açaí seeds, an abundant and low-cost resource, may be a promising source of new treatments and could contribute to reducing the impact of Chagas disease on affected communities.

## 1. Introduction

American trypanosomiasis, commonly known as Chagas disease (CD), is a neglected tropical disease caused by the protozoan *Trypanosoma cruzi* [1]. The disease remains endemic in 21 countries in Latin America but has expanded to non-endemic regions, including the southern United States, Europe, Japan, and Australia. Globally, CD affects approximately 6 million people, with nearly 75 million individuals living in areas at risk of infection, representing a persistent public health challenge [2].

*Trypanosoma cruzi* is a flagellated kinetoplastid protozoan capable of infecting a wide range of host cell types, particularly macrophages, fibroblasts, and epithelial cells [3,4]. Its life cycle alternates between vertebrate and invertebrate hosts and includes three morphologically distinct developmental forms, amastigotes, trypomastigotes, and epimastigotes, which differ in cellular morphology, kinetoplast position, and flagellar organization [5].

Clinically, CD progresses through acute and chronic phases. The acute phase typically begins within 6–10 days after infection and is frequently asymptomatic or associated with mild, nonspecific symptoms [6]. As parasitemia decreases, the disease may evolve into the chronic phase, which can present as an indeterminate form or as cardiac, digestive, or mixed clinical manifestations. Among these, chronic Chagas cardiomyopathy is the most severe and represents the leading cause of CD-related morbidity and mortality, commonly characterized by dilated cardiomyopathy and conduction system abnormalities [7].

The pathogenesis of CD is complex and multifactorial, involving parasite persistence, oxidative stress, and sustained immune activation. During the acute phase, tissue damage is largely associated with parasite replication and a robust inflammatory response. In the chronic phase, residual parasites and parasite-derived antigens maintain low-grade inflammation, contributing to autoimmune responses, progressive myocardial injury, and chronic myocarditis [8,9,10,11,12]. These processes underscore the importance of therapeutic strategies capable of targeting both the parasite and the host inflammatory response.

Currently, CD treatment relies on two nitroheterocyclic drugs, nifurtimox and benznidazole, introduced more than five decades ago [13,14]. Although these drugs are more effective during the acute phase, their efficacy is significantly reduced in chronic infections, with cure rates varying widely [15,16]. Moreover, both treatments are associated with frequent adverse effects, which often lead to treatment interruption, limiting their clinical utility [16,17,18].

In this context, the identification of novel therapeutic agents with improved efficacy and safety profiles remains a priority. Natural products have long been recognized as a valuable source of bioactive compounds, particularly in the development of antiparasitic and anti-inflammatory agents [19,20]. Among these, bioproducts derived from açaí (*Euterpe oleracea* Mart.) seeds have gained attention due to their rich chemical diversity and reported biological activities [21].

Açaí is a palm species native to the Amazon region, whose fruit consists predominantly of a seed accounting for up to 90% of its total mass [22,23,24]. Brazil is currently the world’s largest producer of açaí, generating large volumes of seed residues that are often discarded without adequate treatment, resulting in environmental concerns [25,26]. The reuse of these byproducts represents an opportunity to reduce waste while exploring new sources of biologically active compounds.

Previous studies, including work from our research group, have demonstrated that extracts and fractions from *E. oleracea* seeds exhibit significant antioxidant and anti-inflammatory activities in vitro, without cytotoxic effects on immune cells [21,27,28]. Given the central role of inflammation and oxidative stress in CD pathogenesis, these findings support the investigation of *E. oleracea* seed-derived products as potential therapeutic candidates.

Therefore, the present study aims to evaluate the in vitro anti-*T. cruzi* activity of hydroalcoholic extract and fractions obtained from *E. oleracea* seeds, contributing to the search for novel, safer, and more effective therapeutic strategies for Chagas disease.

## 2. Materials and Methods

### 2.1. Plant Material and Obtention of Hydroalcoholic Extract and Fractions from E. oleracea Seeds

Plant materials were collected in April 2021, at Sepetiba district, Rio de Janeiro, Brazil (22°58′7″ S, 43°41′22″ W) and authenticated by the RBR Herbarium at the Rural Federal University of Rio de Janeiro (voucher specimen RBR 58053). The National Management System granted access to genetic heritage material for Genetic Heritage and Associated Traditional Knowledge (SisGen), the Brazilian Ministry of the Environment, voucher number AAE018A.

Hydroalcoholic extract and fractions of *E. oleracea* seeds were obtained as previously described [28]. After fractionation, all obtained fractions [hexane fraction (HF), dichloromethane fraction (DCMF), ethyl acetate fraction (EAF)] were concentrated under reduced pressure, at 30–40 °C, in a rotary evaporator until complete evaporation of the solvent. The residual fraction, named aqueous fraction (AQF), was lyophilized.

### 2.2. Sample Preparation of Hydroalcoholic Extract and Ethyl Acetate Fraction from E. oleracea Seeds

A total of 1000 µL of methanol (HPLC grade, Tedia, Rio de Janeiro, Brazil) was added to 10 mg of the extract/fraction, previously weighed in a 4 mL vial. The vial was sealed, and the sample was sonicated for 10 min with occasional swirling. Subsequently, the sample was vortexed thoroughly and filtered through a 0.45 µm PTFE filter (Merck Millipore, Darmstadt, Germany) before further analyses into an HPLC vial. The standard catechin solution was prepared using 5 mg of catechin (Sigma-Aldrich, St. Louis, MO, USA) dissolved in 5 mL of methanol in a volumetric balloon, obtaining a final 1 mg/mL solution.

### 2.3. High-Performance Liquid Chromatograph Coupled with a Diode-Array UV-Vis Detector (HPLC-DAD-UV) Analysis

Chromatographic analyses were conducted using high-performance liquid chromatograph coupled with a diode-array UV-Vis detector (HPLC-DAD-UV). The system utilized was a Shimadzu Nexera XR^®^, Kyoto, Japan, liquid chromatograph paired with a Shimadzu UV detector featuring the diode array SPDM20A. The setup included a CBM20A controller, DGU20A degasser, LC20AD binary pump, CTO20A oven, and SILA20A auto-injector. Chromatograms were analyzed using Shimadzu LabSolutions Software Version 5.3. The mobile phase consisted of acidified ultrapure water (pH 3.0, adjusted with anhydrous acetic acid, Merck, Darmstadt, Germany) (A) and acetonitrile (HPLC grade, Tedia, Rio de Janeiro, Brazil) (B), starting at 5% A and increasing to 95% over 80 min. The HPLC column was a silica-based C18 (250 mm × 4.6 mm i.d., 5 µm particle size, ODS Hypersil, Thermo, Waltham, MA, USA). The oven temperature was set at 50 °C, and the injection volume for all analyses was 10 µL.

### 2.4. Characterization of the Hydroalcoholic Extract (HE) and Ethyl Acetate Fraction (EAF) from E. oleracea Seeds by ESI/MS and LC/MS-MS

The extracts were analyzed using direct infusion (ESI-MS/MS) on a Bruker Ion Trap Amazon SL mass spectrometer in negative (ESI-) mode. Samples (2 mg) were dissolved in HPLC-grade methanol using an ultrasonic bath for 20 min. The operating conditions included a 1 µL/min infusion rate, a capillary voltage of 4.0 kV, a source temperature of 100 °C, and a cone voltage ranging from 20 to 40 V. Mass spectra were recorded and interpreted using Bruker Compass Data Analysis 4.2, Bremen, Germany. The LC/MS-MS system consisted of a Shimadzu Nexera UFLC liquid chromatograph connected to a Bruker Daltonics Amazon SL ion trap. The analysis conditions were as follows: (a) room temperature; (b) 100 mm × 2.1 mm × 1.8 μm Aquity HSST3 C18 column, Milford, MA, USA; (c) mobile phase composed by (A) 2.5% acetic acid in water and (B) HPLC-grade acetonitrile. The elution gradient established was 10–85% B in 24 min using a flow rate of 3.0 mL/min and returning to 10% B in 10 min.

### 2.5. Parasites

*Trypanosoma cruzi* Y strain (DTU II) was maintained in vivo in Swiss Webster mice intraperitoneally infected with 1 × 10^6^ parasites. Bloodstream trypomastigotes were obtained from the whole blood by cardiac puncture after animals’ euthanasia at 7 days after infection. Trypomastigotes purification was conducted as described by Araújo-Jorge, 2000 [14]. Epimastigotes forms were obtained after the seed of blood containing the parasites in LIT (Liver Infusion, Triptose) medium [29], supplemented with 10% fetal bovine serum (FBS) (Nova Biotecnologia, São Paulo, Brazil), aseptically, and grown at 28 °C in a B.O.D incubator (MSM-010, MS Mistura, Rio de Janeiro, Brazil). Cell culture-derived trypomastigotes (CCTs) were obtained after infection of VERO cells with blood trypomastigotes (10 parasites/cell). Infected cells were cultured in polystyrene bottles (Corning) with RPMI 1640 medium (Sigma-Aldrich, St. Louis, MO, USA) supplemented with 5% fetal bovine serum (FBS) (Nova Biotecnologia, São Paulo, Brazil), penicillin (100 U/mL) and streptomycin (100 μg/mL) solution (LGC Biotecnologia, São Paulo, Brazil) and incubated at 37 °C and 5% CO_2_ in a humidified incubator (Panasonic—MCO-19AICUV-PA). After 5 days of infection, the culture supernatants were collected and preserved. The cells were washed three times with phosphate-buffered saline (PBS) (Sigma-Aldrich, St. Louis, MO, USA) to completely remove free parasites. The supernatant and the PBS used to wash the cells were transferred to 50 mL conical tubes and centrifuged at 1520× *g* for 10 min at 4 °C. The pellet containing the parasites was resuspended in RPMI medium.

Parasites were quantified in a Neubauer chamber, and their concentrations adjusted for each experiment.

### 2.6. Animals

The Institute of Science and Technology in Biomodels (ICTB/FIOCRUZ) provided the animals under license CEUA Nº: L-007/2021, approved on 8 June 2021. Four-to-six weeks old females or males of Swiss Webster mice were maintained with water and food ad libitum under pathogen-free conditions and controlled temperature in the Carlos Chagas animal facility.

### 2.7. Isolation and Cultivation of Peritoneal Macrophages

Peritoneal macrophages were obtained after injecting 3 mL of a 3% thioglycolate solution (Sigma-Aldrich, St. Louis, MO, USA) in sterile PBS into the animals’ peritoneal cavity. After 72 h, the peritoneal cavity was washed using 10 mL of ice-cold PBS to obtain the cells. Peritoneal lavage was then centrifuged (500 g/5 min/4 °C), and cells were resuspended in RPMI 1640 medium supplemented with 10% FBS, penicillin and streptomycin solution. Cells were quantified in a Neubauer hemocytometer, and viability was verified using trypan blue vital dye.

### 2.8. Cytotoxicity Assay

Peritoneal macrophages (2 × 10^5^ cells/well) were treated with different concentrations of *E. oleracea* seeds hydroalcoholic extract and fractions (500–31.25 µg/mL) and incubated at 37 °C and 5% CO_2_ for 24 h, 48 h, and 72 h. Control wells, untreated cells (culture medium and 1% DMSO), reference drug control wells (cells treated with benznidazole 500–31.25 µg/mL), and blanks were also used. The experiments were conducted in 96-well flat bottom TC-treated microplates (TCP011096) (JetBiofil, Guangzhou, China), in a final volume of 100 µL of complete RPMI 1640 medium. After treatment time, cell viability was analyzed using an MTT colorimetric assay [3-(4,5-dimethylthiazol-2-yl)-2,5-bromidediphenyltetrazolium] [30]. Briefly, 2 h before analysis time, 10 µL of MTT solution (5 mg/mL) was added to each well. Then, after 2 h of incubation, 50 µL of DMSO was added to each well to solubilize the formazan crystals formed by the reduction of the MTT salt. Absorbance was measured in a spectrophotometer (SpectraMax M2, Molecular Devices, Sunnyvale, CA, USA) using a wavelength of 540 nm. The concentration that inhibits 50% of cell growth (CC_50_) was determined by linear regression using the GraphPad Prism 8 software, San Diego, CA, USA.

### 2.9. Anti-T. cruzi Activity

#### 2.9.1. Activity Against Epimastigotes Forms

Epimastigotes forms (2 × 10^5^ parasites/well), in exponential growth phase (2 to 4 days), were seeded in Nunc™ MicroWell™ 96-Well, Nunclon Delta-Treated, Flat-Bottom Microplate (Thermo Fisher Scientific Inc. Waltham, MA, USA) and treated with different concentrations of *E. oleracea* seeds hydroalcoholic extract and fractions (500–15.62 µg/mL), in a final volume of 100 µL of LIT medium. Untreated control wells (containing parasites, culture medium, and 1% DMSO) and reference drug control wells (parasites treated with benznidazole 500–31.2 µg/mL) were also used. The plates were incubated at 28 °C and, after 24 h, 48 h, and 72 h, cell viability was analyzed by manual counting using a Neubauer chamber. The concentration that inhibits 50% of cell growth (IC_50_) was determined.

#### 2.9.2. Activity Against Trypomastigotes Forms

Cell culture-derived trypomastigotes (2 × 10^5^ parasites/well; CCT) or purified blood trypomastigote forms (2 × 10^5^ parasites/well) were plated in 96-well plates, treated with different concentrations of *E. oleracea* seeds hydroalcoholic extract and fractions (500–15.62 µg/mL) in a final volume of 100 µL of DMEM medium and incubated at 37 °C and 5% CO_2_ for 24 h. Untreated control wells (parasites and DMSO 1%) and reference drug control wells (parasites treated with benznidazole 500–31.25 µg/mL) were used. Cell viability was analyzed by manual counting using a Neubauer chamber, and then IC_50_ was determined.

#### 2.9.3. Activity Against Intracellular Amastigotes

Peritoneal macrophages (5 × 10^5^ cells/mL) in complete RPMI medium were seeded in 24-well plates (Costar—3524) containing coverslips and incubated at 37 °C and 5% CO_2_. After 1 h, each well was washed 3 times with sterile PBS to remove non-adherent cells. Then, cells were infected with CCTs (MOI 10:1) and incubated overnight at 34 °C and 5% CO_2_. Then, cells were washed 3 times with sterile PBS to remove non-internalized parasites, treated with different concentrations of *E. oleracea* seeds hydroalcoholic extract and ethyl acetate fraction (500–31.25 µg/mL) in a final volume of 1000 µL of DMEM medium and incubated at 37 °C and 5% CO_2_ for 72 h. Untreated (culture medium and 1% DMSO) and infected control cells and reference drug (benznidazole 500–31.25 µg/mL) treated cells were used. The coverslips were collected at each time point and washed twice in PBS, fixed in BOUIN for 5 min, washed three times with 70% alcohol for 30 min each, and washed with distilled water. After, the coverslips were stained with Giemsa solution (Sigma-Aldrich, St. Louis, MO, USA) for 20 min, washed with distilled water, and dehydrated as follows: two 100% acetone baths, one 70% acetone + 30% xylene bath; one 50% acetone + 50% xylene bath; one 70% acetone + 30% xylene bath and two 100% xylene baths. The coverslips were mounted on slides with Entellan^®^ (Merck, Darmstadt, Germany). The intracellular parasite load was determined by manual counting using light microscopy (100× objective, Axioplan 2 Zeiss^®^ Oberkochen, Alemanha) on three coverslips per infection time. On each coverslip, 200 cells were counted and the number of infected cells and parasites per cell were recorded. The total number of infected cells, the percentage of infection, and the average number of parasites per cell (total number of intracellular amastigotes divided by the percentage of infected cells) was calculated.

### 2.10. Flow Cytometry Assay

Flow cytometry assay was conducted according to the manufacturer’s instructions, using the Dead Cell Apoptosis Kit with Annexin V FITC & Propidium Iodide for Flow Cytometry (Invitrogen, OR, USA).

Briefly, Cell culture-derived trypomastigotes (1 × 10^6^ parasites/mL) were treated with the respective IC_50_ of hydroalcoholic extract and ethyl acetate fraction of *E. oleracea* seeds in DMEM medium and incubated for 24 h at 37 °C and 5% CO_2_. Heat-killed *Trypanosoma cruzi* trypomastigotes (60 °C for 60 min) were used as a positive control, while untreated parasites were used as the negative control. After the incubation, the parasites were centrifuged (1520× *g*/10 min), washed in cold phosphate-buffered saline (PBS), resuspended in a 1× annexin-binding buffer and subsequently incubated with Annexin V FITC and PI at room temperature for 15 min. Measurements were acquired using a CytoFlex flow cytometer (Beckman Coulter Life Sciences, Indianapolis, IN, USA) with fluorescence emission at 530 nm (e.g., FL1) and 575 nm (e.g., FL3). CytExpert software version 2.6 (Beckman Coulter Life Sciences, Indianapolis, IN, USA) was used for flow cytometry analyses.

### 2.11. Statistical Analysis

Analyses were performed using GraphPad Prism 8.2.1 software (GraphPad Software, San Diego, CA, USA). The data were expressed by mean ± standard deviation (SD) from independent experiments performed in triplicate and analyzed statistically by Kruskal–Walli’s test, followed by multiple comparisons correction using the Benjamini–Hochberg. Differences were considered significant when *p* < 0.05. IC_50_ and CC_50_ were calculated by linear regression.

## 3. Results and Discussion

### 3.1. Hydroalcoholic Extract and the Ethyl Acetate Fraction from E. oleracea Seeds Are Rich in Catechins and Procyanidins

The extracts were characterized using ESI/MS and HPLC/MS-MS analyses. Compounds were tentatively identified by comparing their molecular ions with data reported in the literature. The chemical profiles of the hydroalcoholic extract and the ethyl acetate fraction of *E. oleracea* seeds are presented in Table 1, while the profiles of the remaining fractions (hexane, dichloromethane, and aqueous) have been previously reported by our group [28]. Overall, the chemical compositions of the extract and fractions of *E. oleracea* seeds are consistent those described in earlier studies [31,32].

**Figure 1:**
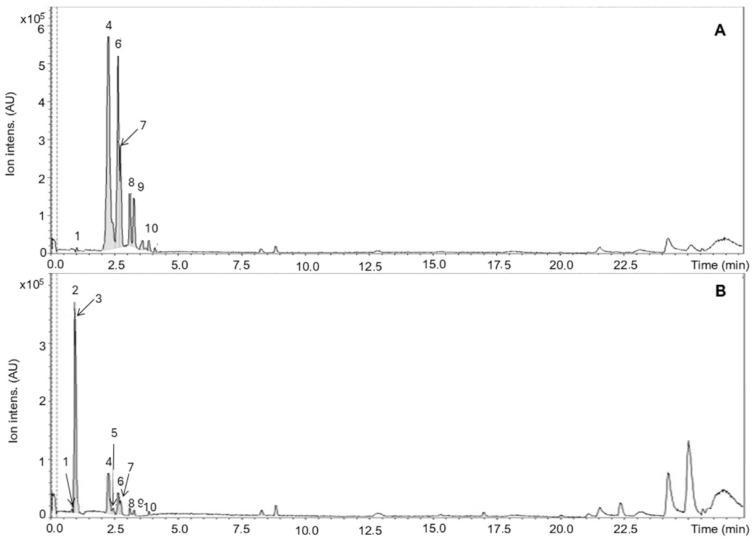
HPLC–MS/MS chromatograms of *Euterpe oleracea* seed. (**A**): Ethyl acetate fraction (EAF). (**B**): Hydroalcoholic extract (HE). Peaks are labeled according to Table 1, with retention times (Rt, min) and tentative identifications. The y-axis represents ion intensity (AU). Differences between panels highlight the relative abundance of catechins and procyanidins in EAF versus HE.

Figure 1 shows the HPLC–MS/MS chromatograms of the hydroalcoholic extract and ethyl acetate fraction derived from *E. oleracea* seeds. In both chromatograms, peaks corresponding predominantly to polar compounds were observed, indicating their high abundance in the analyzed samples. Data obtained from ESI/MS analyses were essential to corroborate the presence of specific compounds previously detected by LC–MS/MS. Both samples exhibited signals corresponding to catechin (*m*/*z* 288.91), procyanidin dimer B1 (*m*/*z* 577.06), and petunidin 3-O-(6″-p-coumaroyl-glucoside) (*m*/*z* 623.25).

**Figure 2:**
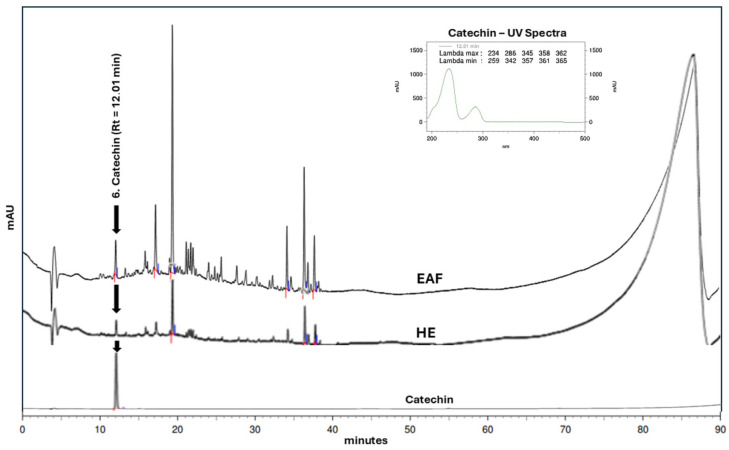
Comparative HPLC analysis of the hydroalcoholic extract and ethyl acetate fraction of *Euterpe oleracea* seeds with the catechin standard.

To confirm the presence of catechin in both samples, HPLC analyses were performed by comparing the chromatographic profiles of the hydroalcoholic extract and the ethyl acetate fraction with that of a catechin reference standard (Figure 2). These data, together with the mass spectrometry results described above, confirmed the presence of catechin in both the hydroalcoholic extract (HE) and the ethyl acetate fraction (EAF). Moreover, differences in signal intensity between the samples suggest a higher catechin content in the ethyl acetate fraction.

Overall, the chromatographic profiles of the analyzed samples were highly similar, differing mainly in the relative concentrations of specific constituents.

As reported by our group and other authors, catechins and procyanidins are the predominant compounds in bioproducts derived from açaí seeds [28,32,33,34,35]. These molecules belong to the flavonoid class of polyphenols and are widely recognized for their diverse biological activities. Previous studies have demonstrated that catechins and procyanidins exhibit strong antioxidant properties, contributing to the regulation of oxidative stress, a process implicated in both parasite survival and host–parasite interactions during *T. cruzi* infection [36]. In addition, their anti-inflammatory effects have been extensively documented, including the modulation of proinflammatory mediators. This activity is particularly relevant in the context of Chagas disease, in which inflammation plays a central role in tissue damage and disease progression [37]. Collectively, these properties place catechins and procyanidins as promising bioactive molecules in the search for novel antiparasitic agents or adjuvants for Chagas disease therapy.

Within this antiparasitic framework, the evaluation of potential synergistic, additive, or antagonistic interactions between catechin and procyanidin becomes particularly relevant. Combination-based approaches have gained increasing attention as strategies to enhance therapeutic efficacy, reduce required drug concentrations, and potentially mitigate toxicity and resistance associated with current chemotherapeutic options [38]. Natural products often act on multiple cellular targets, and the combined effects of structurally related polyphenols may potentiate parasite killing or modulate stress-response pathways in *T. cruzi*. Accordingly, future studies should investigate these interactions using established pharmacological tools, such as isobologram analysis and the determination of the fractional inhibitory concentration index (FICI), which are widely applied to assess compound interactions in antiparasitic and antimicrobial research [38,39]. Such analyses would enable a more refined evaluation of whether catechins and procyanidins act synergistically against *T. cruzi*, thereby supporting the rational development of optimized combinations or phytochemical-based therapeutic strategies for Chagas disease.

### 3.2. Treatment with Hydroalcoholic Extract and Fractions of E. oleracea Exhibits Low or No Toxicity to Peritoneal Macrophages

Assessing the cell viability of extracts and their fractions is essential for elucidating potential cytotoxic effects [40]. In this study, the cytotoxic effects of the hydroalcoholic extract and the ethyl acetate, aqueous, hexane, and dichloromethane fractions obtained from açaí seeds was evaluated in peritoneal macrophages derived from Swiss Webster mice. Murine peritoneal macrophages were selected as a biologically relevant primary cell model, as these immune cells play a central role in *Trypanosoma cruzi* infection and parasite control in vivo, particularly through nitric oxide-mediated mechanisms. Moreover, their use enables the assessment of intracellular amastigote survival and host–parasite interactions, thereby complementing data generated using immortalized cell lines.

No significant cytotoxicity was observed after 24 or 48 h of treatment, with CC_50_ values exceeding 500 µg/mL for all extracts and fractions tested. After 72 h of exposure, the hydroalcoholic extract exhibited a CC_50_ of 433 µg/mL, while the aqueous fraction presented a CC_50_ of 467.5 µg/mL, indicating a time-dependent increase in cytotoxicity toward peritoneal macrophages (Table 2).

Comparable findings were reported by Filho et al. (2023) [31], who evaluated the cytotoxic effects of a hydroalcoholic extract from açaí seeds on CCD-1072Sk fibroblasts. In that study, cytotoxicity was both time- and concentration-dependent, with significant effects observed only after a 72 h exposure [31].

In contrast, the ethyl acetate, hexane, and dichloromethane fractions did not exhibit cytotoxic effects), even after 72 h of treatment, with CC_50_ values remaining above 500 µg/mL (Table 2). These results are consistent with those reported by Xavier et al. (2021) [32], who demonstrated the absence of cytotoxicity of the ethyl acetate fraction in RAW 264.7 macrophages following 48 h of exposure [32].

Supporting these observations, Barros Dias et al. (2024) [41], published a comprehensive review analyzing 72 studies on the biological activities and pharmacological properties of açaí seeds, concluding that these bioproducts generally do not exhibit cytotoxic effects and present a promising safety profile. Nevertheless, the authors emphasized the need for further clinical investigations to elucidate the bioavailability, metabolic pathways, and pharmacokinetic properties of açaí seed extracts. Such data are essential for accurately assessing their safety and therapeutic potential [41].

Although the hydroalcoholic extract and fractions of *E. oleracea* seeds did not induce marked cytotoxicity in peritoneal macrophages under the experimental conditions employed in this study, this favorable safety profile should be interpreted with caution. Phenolic compounds, including catechins and proanthocyanidins, which are among the main constituents reported for açaí seeds, have been widely documented to exert dose-dependent cytotoxic effects in various mammalian cell types, particularly epithelial and cancer cell lines. Therefore, the lack of cytotoxicity observed in macrophages does not necessarily imply generalized cellular safety. In this context, further investigations addressing the pharmacokinetics, bioavailability, metabolic fate, and comprehensive toxicity profiles of *E. oleracea* seed-derived bioproducts in both in vitro and in vivo models are warranted to better define their therapeutic window and translational potential.

### 3.3. Antiparasitic Activity of the Hydroalcoholic Extract and Fractions from Euterpe oleracea Seeds

The antiparasitic activity of the hydroalcoholic extract, ethyl acetate fraction, and the aqueous fraction obtained from açaí seeds was evaluated against epimastigotes, bloodstream tripomastigotes, cell culture-derived trypomastigotes (CCTs) and intracellular amastigote forms of *T. cruzi* (Y strain).

Regarding epimastigote forms, all samples exhibited weak and time-dependent inhibitory effects, with no detectable trypanocidal activity after 24 or 48 h of exposure (IC_50_ > 500 μg/mL). Only after prolonged incubation (72 h) did the hydroalcoholic extract and the ethyl acetate fraction show a reduction in IC_50_ values, with the ethyl acetate fraction presenting the lowest IC_50_ (199.4 ± 1.43 μg/mL). Nevertheless, all samples were markedly less active than benznidazole, and overall activity against epimastigotes was low, as shown in Table 3.

In contrast, both hydroalcoholic extract and the ethyl acetate fraction exhibited trypanocidal activity against bloodstream trypomastigotes of *T. cruzi* Y strain after 24 h of exposure, with IC_50_ values of 308.30 µg/mL and 115.12 µg/mL, respectively (Table 4). The ethyl acetate fraction showed higher activity against this infective stage, and a superior selectivity index compared with the hydroalcoholic extract.

Evaluation of activity against cell culture-derived trypomastigote forms demonstrated that both the hydroalcoholic extract and its fractions exerted inhibitory effects. Notably, the ethyl acetate fraction displayed the most potent activity, with the lowest IC_50_ (123.46 ± 2.52 µg/mL) and the highest selectivity index (SI = 4.05) for this developmental stage, as detailed in Table 5.

Intracellular amastigote forms were treated with the hydroalcoholic extract and the ethyl acetate fraction for 72 h. The hydroalcoholic extract exhibited a lower IC_50_ value (40.04 ± 1.05 μg/mL) and a higher selectivity index (SI = 10.81) than the ethyl acetate fraction (IC_50_ = 67.02 ± 1.15 μg/mL; SI = 7.46), indicating selective targeting of *T. cruzi* by the hydroalcoholic extract (Table 6). As expected, benznidazole showed low IC_50_ values across all infective stages.

**Figure 3:**
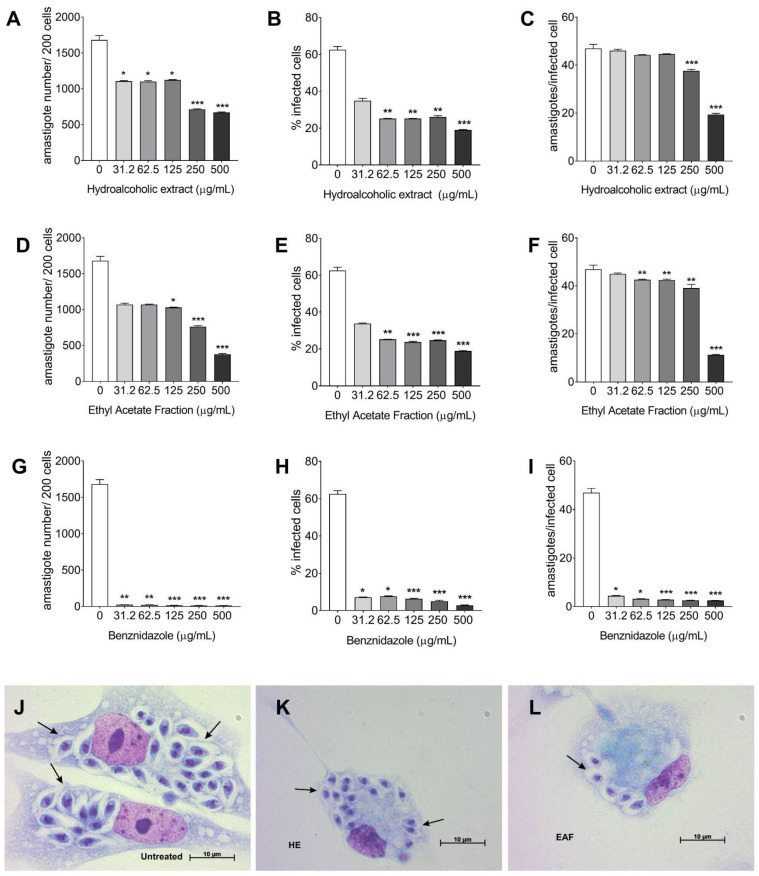
Infection parameters of peritoneal macrophages infected by *T. cruzi* Y strain and treated for 72 h with hydroalcoholic extract (**A**–**C**), ethyl acetate fraction (**D**–**F**) from *E. oleracea* seeds, and benznidazole (**G**–**I**). Light microscopy images of infected and untreated cells, treated with hydroalcoholic extract and with the ethyl acetate fraction of *E. oleracea* seeds at 500 μg/mL (**J**–**L**). Giemsa staining, 100x objective. Intracellular amastigotes within peritoneal macrophages (black arrows). The data represent the mean ± standard deviation from three experiments conducted in triplicate. *p*-value *** < 0.001, ** < 0.002, * < 0.033, compared to infected and untreated cells, by the Kruskal–Wallis test, followed by multiple comparisons correction using the Benjamini–Hochberg.

Analysis of infection parameters revealed that treatment with both the hydroalcoholic extract, and the ethyl acetate fraction resulted in a marked reduction in the total number of intracellular amastigotes and a decreased percentage of infected cells after 72 h at all tested concentrations (Figure 3A–F). Notably, the ethyl acetate fraction demonstrated superior efficacy in reducing both the total number of amastigotes and the mean number of amastigotes per infected cell after 72 h, particularly at 500 µg/mL (Figure 3F). Benznidazole treatment also significantly reduced all evaluated infection parameters.

The therapeutic potential of natural products against parasitic diseases has been extensively documented. Numerous studies have demonstrated trypanocidal activity of plant-derived bioproducts and have associated this activity with the presence of phenolic compounds, particularly flavonoids [42]. In this context, the hydromethanolic extract of *Plinia cauliflora* Mart. Kausel (jabuticaba) has been reported to exhibit anti-*Trypanosoma cruzi* activity, attributed to its phenolic with antioxidant, antimicrobial, and antiparasitic properties. Phenolic compounds are known to interact with membrane lipids, proteins, and enzymes, leading to enzyme inactivation, mitochondrial dysfunction, and inhibition of metabolic pathways essential for parasite survival and differentiation [43].

Similarly, Castañeda et al. (2021) [44] evaluated the trypanocidal activity of ethanolic extracts obtained from six plant species against epimastigote and trypomastigote forms of *T. cruzi* (Colombiana 058PUJ), while assessing cytotoxicity in peripheral blood mononuclear cells (PBMCs). Extracts from *Ageratina vacciniaefolia*, *Clethra fimbriata*, and *Siparuna sessiliflora* exhibited significant antiparasitic activity against with low cytotoxicity. Chemical profiling revealed a predominance of flavonoids and terpenoids, likely contributing to the observed bioactivity [44].

Additional evidence supporting the antiparasitic role of phenolic comes from studies on vestitol isolated from Brazilian red propolis, which demonstrated a strong correlation between flavonoid content and efficacy against *T. cruzi*, reinforcing the therapeutic potential of these phytochemicals for Chagas disease (CD) [40]. Likewise, flavonoids isolated from the aerial parts of *Delphinium staphisagria* showed pronounced antiproliferative activity against epimastigote and amastigote forms of *T. cruzi* in both in vitro and in vivo models [45]. This activity has been largely attributed to catechins, identified as major constituents of active fractions [46].

Catechin-class phenolic compounds have been consistently reported to exert antiparasitic activity against trypanosomatids, including *T. cruzi*. Their mechanisms of action are primarily linked to interference with essential cellular processes, particularly those involved in redox homeostasis. Given the unique antioxidant metabolism of *T. cruzi*, these compounds may increase parasite susceptibility to oxidative stress, leading to mitochondrial dysfunction, reduced ATP production, and parasite death. Gallated catechins have also been shown to interact directly with key parasitic enzymes. Although most mechanistic studies have been performed in *Leishmania* species, the functional conservation of enzymes such as trypanothione reductase among trypanosomatids supports the hypothesis that similar mechanisms may operate in *T. cruzi* [46,47].

Despite these promising findings, most available studies remain limited to in vitro assays, underscoring the need for further investigations to better elucidate the therapeutic potential of these compounds. Notably, to date, no studies have evaluated the trypanocidal activity of açaí seed extracts or their fractions, highlighting the innovative aspect of the present research.

Although epimastigote forms of *T. cruzi* are not clinically relevant targets for drug discovery in Chagas disease, since this developmental stage occurs exclusively in the digestive tract of triatomine vectors, epimastigotes remain useful for preliminary screening due to their ease of cultivation and experimental reproducibility [40,44,48]. Moreover, targeting this stage may contribute indirectly to transmission control by reducing parasite burden in triatomine vectors [49].

Taken together, these findings indicate that açaí seed extracts and fractions display limited activity against epimastigote forms of *T. cruzi*, reinforcing that this developmental stage is not predictive of therapeutic efficacy. Therefore, the interpretation of antiparasitic potential in this study is primarily based on results obtained against infective trypomastigote forms and intracellular amastigotes, which are directly relevant to mammalian infection and disease progression.

According to the Drugs for Neglected Diseases initiative (DNDi), promising drug candidates for Chagas disease should exhibit activity against both intracellular amastigotes and trypomastigotes, with a selectivity index greater than 10 [2]. The inclusion of both evolutionary forms in drug discovery studies is essential due to their central role in parasite infectivity and persistence within vertebrate hosts [50].

When screening trypanocidal activity against trypomastigote forms, it is critical to consider that these parasites do not replicate and exhibit a limited lifespan. As a result, assays must be conducted within a 24 h period to prevent loss of viability or differentiation into epimastigote-like forms. Typically, bloodstream trypomastigotes and those derived from cell cultures—whether genetically modified or not—are employed. These infective stages predominate during the acute phase of Chagas disease [50]. The experimental design using these evolutive forms may underestimate the activity of slow-acting compounds, whose trypanocidal effects might require longer exposure times to become evident. Accordingly, while the 24 h exposure provides a robust and standardized assessment of acute trypanocidal activity, additional assays using alternative experimental approaches may be necessary in future studies to better evaluate delayed or cumulative antiparasitic effects.

While the hydroalcoholic extract and fractions from açaí seeds showed measurable activity against both cell culture-derived and bloodstream trypomastigote forms, all selectivity indices remained below the recommended threshold for progression in the Chagas disease drug discovery pipeline [2]. These results suggest that these bioproducts may be classified as early-stage hits, warranting further investigation through bioactivity-guided fractionation and optimization to enhance potency and selectivity [2].

An essential aspect of CD drug screening is the use of experimental models that accurately reproduce the interactions between *T. cruzi* and host immune cells. Targeting the intracellular amastigotes of *T. cruzi* is critical, considering their persistence in infected individuals throughout the disease’s progression. Additionally, the chronic phase of CD remains a significant challenge, as effective therapeutic options are lacking at that stage [50]. Assays targeting the intracellular amastigotes in macrophages are particularly informative, as macrophage activation and nitric oxide production play central roles in parasite control [51]. In this context, treatment of *T. cruzi*-infected peritoneal macrophages demonstrated that the hydroalcoholic extract achieved a selectivity index greater than 10, indicating selective targeting of intracellular amastigotes.

Although a favorable selectivity index was observed only for the hydroalcoholic extract against intracellular amastigotes, complementary studies, including in vivo assays, pharmacokinetic analyses, and toxicity profiling, are necessary to further define the therapeutic potential of *Euterpe oleracea* seed-derived bioproducts against *T. cruzi*.

### 3.4. The Treatment with Hydroalcoholic Extract and the Ethyl Acetate Fraction of E. oleracea Seeds Reduces the Cell Viability of Cell-Derived Culture Trypomastigotes Inducing Death by Late Apoptosis

To investigate the cellular effects induced by the hydroalcoholic extract and the ethyl acetate fraction of *Euterpe oleracea* seeds on culture-derived *Trypanosoma cruzi* trypomastigotes, flow cytometry analysis using Annexin V-FITC and propidium iodide (PI) staining was employed as an exploratory approach. This strategy allowed the characterization of alterations in plasma membrane integrity and cell death–associated phenotypes, providing mechanistic insight into the cellular impact of these candidate antiparasitic compounds. The combined staining enables discrimination between parasites exhibiting phosphatidylserine (PS) externalization and those with advanced membrane permeabilization, which are commonly interpreted as early and late stages of cell death in eukaryotic cells.

**Figure 4:**
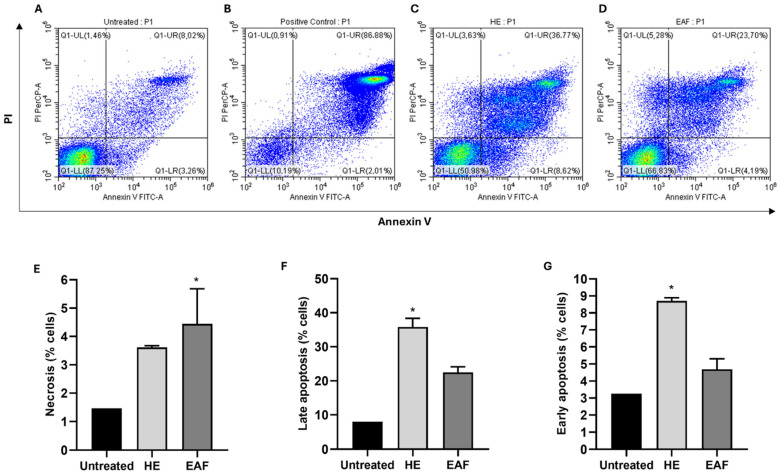
Annexin V/PI analysis of cell culture-derived trypomastigote forms of *T. cruzi* Y strain, treated with the IC_50_ value of the hydroalcoholic extract (253 μg/mL) and ethyl acetate fraction (123 μg/mL) of *E. oleracea* seeds followed by Annexin V-FITC and PI staining. (**A**) Untreated trypomastigotes; (**B**) Positive control; (**C**) Trypomastigotes treated with the hydroalcoholic extract (HE); (**D**) Trypomastigotes treated with the ethyl acetate fraction (EAF); (**E**–**G**) Statistical differences in the percentage of early apoptotic (Annexin V+), necrotic (PI+), and late apoptotic (Annexin V+PI+) trypomastigotes among untreated parasites, parasites treated with the hydroalcoholic extract, and those treated with the ethyl acetate fraction. Results are expressed as mean ± standard deviation (SD) from experiments performed in triplicate compared to untreated parasites, based on the Kruskal–Wallis test adjusted by Benjamini and Hochberg’s method. * *p* < 0.033.

Treatment with 253 µg/mL of the hydroalcoholic extract for 24 h resulted in a pronounced reduction in parasite viability, decreasing from 87% in untreated controls to 50%. Within the treated population, 8.71% of parasites were Annexin V–positive, 3.61% were exclusively PI–positive, and a substantial fraction (35.81%) exhibited dual Annexin V/PI staining, indicative of extensive membrane damage. Similarly, exposure to 123 µg/mL of the ethyl acetate fraction for 24 h reduced viability from 87% to 67%, with 4.69% Annexin V–positive cells, 4.44% PI–positive cells, and 22.48% dual-stained parasites (Table 7; Figure 4A–F).

Although Annexin V/PI cytometry is widely used to classify apoptotic and necrotic cell populations in metazoans [52], its interpretation in trypanosomatids requires particular caution. Genomic and biochemical studies have demonstrated that *Trypanosoma* and *Leishmania* species lack several key components of the canonical apoptotic machinery, including caspases, Bcl-2 family proteins, and death receptors, which are central to apoptosis signaling in higher eukaryotes [53,54,55]. Consequently, Annexin V binding and PI uptake in *T. cruzi* trypomastigotes should be interpreted primarily as indicators of PS externalization and loss of plasma membrane integrity, respectively, rather than as definitive evidence of apoptosis.

Despite these limitations, the use of apoptosis-like markers remains a valuable phenotypic tool to assess drug-induced cellular damage in trypanosomatids, particularly when interpreted within an appropriate conceptual framework. Similar Annexin V/PI profiles, characterized by a predominance of dual-stained parasites suggestive of late-stage or irreversible cellular damage, have been reported for other natural products with trypanocidal activity, including the ethanolic extract of *Physalis angulata*, which induced extensive membrane disruption and loss of parasite viability in *T. cruzi* [56,57]. In this context, our findings demonstrate that both the hydroalcoholic extract and the ethyl acetate fraction of *E. oleracea* seeds significantly compromise parasite viability, with a marked accumulation of Annexin V/PI–positive populations.

Taken together, these results support the conclusion that the tested treatments induce severe and irreversible cellular damage consistent with apoptosis-like or late-stage cell death phenotypes in *T. cruzi* trypomastigotes. While the underlying molecular pathways cannot be definitively classified as apoptotic, the observed membrane alterations and loss of parasite viability reinforce the antiparasitic potential of *E. oleracea* seed-derived products. These findings warrant further investigation using complementary approaches, including assays of mitochondrial membrane potential and alternative markers of regulated cell death, to better elucidate the mechanisms involved.

## 4. Conclusions

The present study provides the first comprehensive evaluation of the chemical composition, cytotoxicity, and trypanocidal activity of açaí (*Euterpe oleracea*) seed extracts and fractions against multiple developmental stages of *Trypanosoma cruzi*. Chemical analyses confirmed the predominance of phenolic compounds, especially catechins and procyanidins, which are widely associated with antiparasitic, antioxidant, and anti-inflammatory activities.

Biological assays demonstrated that although the extracts and fractions exhibited limited activity against epimastigote forms, they displayed measurable and biologically relevant trypanocidal effects against infective trypomastigote stages and intracellular amastigotes, in addition to a favorable cytotoxic profile in peritoneal macrophages. This bioactivity may be attributed to the flavonoid compounds present in açaí seeds, as supported by existing literature. Importantly, the hydroalcoholic extract showed selective activity against intracellular amastigotes, achieving a selectivity index greater than 10, a critical benchmark for progression in Chagas disease drug discovery pipelines. These findings underscore the importance of prioritizing clinically relevant parasite stages when evaluating antiparasitic candidates.

Both the hydroalcoholic extract and the ethyl acetate fraction from açaí seeds induced the death of *Trypanosoma cruzi* trypomastigotes, predominantly through late apoptosis-like pathways. Overall, the results indicate that açaí seed-derived bioproducts represent promising early-stage hits with potential applicability in Chagas disease therapy. Nevertheless, further studies are required to advance these findings, including bioactivity-guided fractionation, mechanistic investigations, pharmacokinetic and toxicity assessments, and in vivo efficacy studies. Such approaches will be essential to fully define the therapeutic window and translational potential of *E. oleracea* seed extracts as novel antiparasitic agents.

## Figures and Tables

**Table 1:** Tentative identification of the hydroalcoholic extract and ethyl acetate fraction of *E. oleracea* seeds by mass spectrometry.

	Rt	Molecular Formula	*m*/*z*	Tentative Identification	HE	EAF
** 1 **	0.8	-	232.9697	n.i.	+	−
** 2 **	0.9	-	683.2074	n.i.	+	−
** 3 **	1.0	-	377.0764	n.i.	+	+
** 4 **	2.3	C_30_H_26_O_12_	577.1155	Procyanidin dimer B1	+	+
** 5 **	2.5	-	720.1404	n.i.	+	−
** 6 **	2.6	C_15_H_14_O_6_	289.0640	(+)-Catechin	+	+
** 7 **	2.7	C_30_H_26_O_12_	577.1192	Procyanidin dimer B1	+	+
** 8 **	3.1	C_15_H_14_O_6_	289.0649	(−)-Epicatechin	+	+
** 9 **	3.3	C_30_H_26_O_12_	577.1187	Procyanidin dimer B1	+	+
** 10 **	3.8	C_31_H_29_O_14_	623.1399	Petunidin 3-O-(6″-p-coumaroyl-glucoside)	+	+

Note: Rt = retention time, *m*/*z* = mass/charge ratio. The signs (+) and (−) indicate the presence or absence of the compound. HE = hydroalcoholic extract; EAF = ethyl acetate fraction; n.i. = not identified.

**Table 2:** Cytotoxicity of the hydroalcoholic extract and the ethyl acetate, aqueous, hexane, and dichloromethane fractions of *E. oleracea* seeds on peritoneal macrophages.

*E. oleracea* Seeds	Cytotoxicity CC_50_ (µg/mL)		
	24 h	48 h	72 h
Hydroalcoholic extract	>500	>500	433 ± 1.31
Ethyl acetate fraction	>500	>500	>500
Aqueous fraction	>500	>500	467.5 ± 1.21
Hexanic fraction	>500	>500	>500
Dichloromethane fraction	>500	>500	>500
Benznidazole	>500	>500	>500

Data represents the mean ± standard deviation of the three independent experiments performed in triplicate. CC_50_ was calculated by linear regression. CC_50_: cytotoxic concentration for 50% of the cells.

**Table 3:** The activity of the hydroalcoholic extract, ethyl acetate fraction, and aqueous fraction from *E. oleracea* seeds on epimastigote forms of *T. cruzi* Y strain.

*Euterpe oleracea* Seeds	24 h		48 h		72 h	
	IC_50_ (μg/mL)	SI	IC_50_ (μg/mL)	SI	IC_50_ (μg/mL)	SI
Hydroalcoholic extract	>500	n.d.	>500	n.d.	482 ± 1.40	0.89
Ethyl acetate fraction	>500	n.d	>500	n.d.	199.4 ± 1.43	>2.50
Aqueous fraction	>500	n.d.	>500	n.d.	>500	0.51
Benznidazole	<31.25	n.d.	<31.25	n.d.	<31.25	n.d.

Data represent the mean ± standard deviation from three independent experiments conducted in triplicate. IC_50_ was calculated by linear regression. IC_50_: concentration required to inhibit 50% of parasites; SI: selectivity index for epimastigote forms (SI = CC_50_/IC_50_). n.d. = not done.

**Table 4:** Activity of the hydroalcoholic extract and ethyl acetate fraction from *E. oleracea* seeds on bloodstream trypomastigote forms of *T. cruzi* Y strain after 24 h of treatment.

*Euterpe oleracea* Seeds	*T. cruzi* Y Strain IC_50_ (µg/mL)	
	Bloodstream Trypomastigotes	SI
Hydroalcoholic extract	308.30 ± 1.11	1.62
Ethyl acetate fraction	115.12 ± 1.09	4.34
Benznidazole	<31.25	n.d.

Data represent the mean ± standard deviation from three independent experiments conducted in triplicate. IC_50_ was calculated by linear regression. IC_50_: drug concentration that reduces 50% of the number of treated parasites; SI: selectivity index for bloodstream trypomastigote forms (SI = CC_50_/IC_50_). n.d. = not done.

**Table 5:** Effect of the hydroalcoholic extract and fractions from *E. oleracea* seeds on cell culture-derived trypomastigote forms (CCTs) of *T. cruzi* Y strain after 24 h of treatment.

*Euterpe oleracea* Seeds	*T. cruzi* Y Strain IC_50_ (µg/mL)	
	CCTs	SI
Hydroalcoholic extract	252.83 ± 2.95	1.97
Ethyl acetate fraction	123.46 ± 2.52	4.05
Aqueous fraction	258.27 ± 2.78	1.93
Hexanic fraction	494.78 ± 0.46	1.01
Dichloromethane fraction	353.72 ± 1.79	1.41
Benznidazole	<31.25	n.d.

Data represent the mean ± standard deviation from three independent experiments conducted in triplicate. IC_50_ was calculated by linear regression. IC_50_: drug concentration that reduces 50% of the number of treated parasites; SI: selectivity index for cell-culture-derived trypomastigote forms (CCTs) (SI = CC_50_/IC_50_). n.d. = not done.

**Table 6:** Effect of the hydroalcoholic extract and ethyl acetate fraction from *E. oleracea* seeds on intracellular amastigote forms of *T. cruzi* Y strain.

*Euterpe oleracea* Seeds	72 h	
	IC_50_ (μg/mL)	SI
Hydroalcoholic extract	40.04 ± 1.05	10.81
Ethyl acetate fraction	67.02 ± 1.15	7.46
Benznidazole	<31.25	n.d.

Data represent the mean ± standard deviation from three experiments conducted in triplicate. IC_50_ was calculated by linear regression. IC_50_: concentration required to inhibit 50% of the parasites; SI: selectivity index for intracellular amastigote forms (SI = CC_50_/IC_50_). n.d. = not done.

**Table 7:** *E. oleracea* seed hydroalcoholic extract and ethyl acetate fraction induced cell derived trypomastigotes death, as revealed by Annexin V and/or PI labeling.

*Euterpe oleracea* Seeds	Percentage (%)		
	Annexin V+	PI+	Annexin V+/PI+
Hydroalcoholic extract	8.71 ± 0.19	3.61 ± 0.06	35.81 ± 2.55
Ethyl acetate fraction	4.69 ± 0.62	4.44 ± 1.24	22.48 ± 1.66
Untreated	3.26 ± 0.05	1.46± 0.02	8.02 ± 0.15

Percentage of Early Apoptotic (Annexin V+), Necrotic (PI+), and Late Apoptotic (Annexin V+/PI+) Trypomastigotes from Untreated Parasites and Those Treated with Hydroalcoholic Extract, Ethyl Acetate Fraction. Data presented as mean ± standard deviation (SD) from experiments performed in triplicate.

## Data Availability

The original contributions presented in this study are included in the article. Further inquiries can be directed to the corresponding authors.
